# Non-target estrogenic screening of 60 pesticides, six plant protection products, and tomato, grape, and wine samples by planar chromatography combined with the planar yeast estrogen screen bioassay

**DOI:** 10.1007/s00216-023-04605-x

**Published:** 2023-03-06

**Authors:** Annabel Mehl, Sophia Seiferling, Gertrud E. Morlock

**Affiliations:** Institute of Nutritional Science, Chair of Food Science, and Interdisciplinary Research Center, Justus Liebig University Giessen, Heinrich-Buff-Ring 26-32, 35392 Giessen, Germany

**Keywords:** Residue analysis, Bioautography, Planar yeast estrogen screen, Endocrine-disrupting compounds, Food safety

## Abstract

For non-target residue analysis of xenoestrogens in food, sophisticated chromatographic–mass spectrometric techniques lack in biological effect detection. Various in vitro assays providing sum values encounter problems when opposing signals are present in a complex sample. Due to physicochemical signal reduction, cytotoxic or antagonistic effect responses, the resulting sum value is falsified. Instead, the demonstrated non-target estrogenic screening with an integrated planar chromatographic separation differentiated opposing signals, detected and prioritized important estrogenic compounds, and directly assigned tentatively the responsible compounds. Sixty pesticides were investigated, ten of which showed estrogenic effects. Exemplarily, half-maximal effective concentrations and 17β-estradiol equivalents were determined. Estrogenic pesticide responses were confirmed in six tested plant protection products. In food, such as tomato, grape, and wine, several compounds with an estrogenic effect were detected. It showed that rinsing with water was not sufficient to remove selected residues and illustrated that, though not usually performed for tomatoes, peeling would be more appropriate. Though not in the focus, reaction or breakdown products that are estrogenic were detected, underlining the great potential of non-target planar chromatographic bioassay screening for food safety and food control.

**Graphical Abstract:**

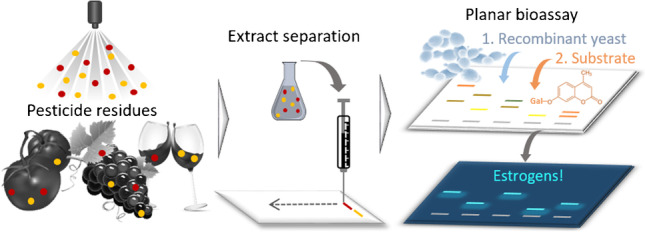

**Supplementary information:**

The online version contains supplementary material available at 10.1007/s00216-023-04605-x.

## Introduction

Plant protection products (PPPs) are used worldwide in agriculture to protect plants against harmful organisms, influence the life processes of plants, preserve plant products, destroy undesired plants, and prevent undesired growth of plants [1]. They contain active substances (referred to as pesticides), safeners (reducing phytotoxic effects on certain plants), or synergists (enhancing pesticide activity) for at least one of the mentioned purposes [1]. Besides their positive effects, they may be harmful to nature in general by disturbing the natural balance, and to humans if foods are consumed that contain residues of these pesticides. For consumer protection, maximum residue levels (MRLs) at the 0.01-mg/kg level were laid down by the European Union (EU) for a large number of different food products [2, 3]. Below these levels, consumption is considered safe. However, for some pesticides and their transformation products, xenoestrogenic activity has been reported frequently [4–9]. Xenoestrogens can interact with the human estrogen receptors hERα and hERβ and thus disturb the normal reproductive process [10], leading, for example, to low sperm count and adverse pregnancy outcomes [11]. Combined effects of multiple xenoestrogens are known to occur even at doses well below the no-observed adverse effect level [10], and thus also below the MRL of a single xenoestrogen, impairing food safety.

Screening for estrogenic activity is largely performed by in vitro bioassays. Most common are cell proliferation assays, such as the estrogen (E)-screen, and reporter gene assays, such as the ER-chemical-activated luciferase gene expression (CALUX) assay and the liquid yeast estrogen screen (lYES) [12]. In the lYES bioassay, recombinant yeast cells are used whose DNA contains sequences for the hER and the reporter gene *lacZ* [13]. If agonists bind to the estrogen-responsive element that is fused to the *lacZ* gene, the latter is expressed and produces β-galactosidase [13]. This enzyme can react with different galactose-containing substrates, such as 4-methylumbelliferyl-β-d-galactopyranoside (MUG), which is cleaved into the blue fluorescent 4-methylumbelliferone (MU). As the resulting bioassay end-product signal, the MU-blue fluorescence indicates the presence of estrogenic compounds [14]. The biological detection is advantageous because it places the analytical focus on a specific effect (wide non-target view) instead of a compound or substance class (limited target view). However, the lYES is carried out in vitro in microtiter plates [15], and therefore, it only provides a sum value result for a multicomponent sample. Unfortunately, in vitro assays cannot distinguish or differentiate between compounds providing opposite signals due to physicochemical signal reduction, cytotoxic or antagonistic effect responses. In case of opposing signals or effects being present in a complex sample, in vitro assays can lead to a falsified sum value result and thus false conclusions drawn. For example, the estrogenic compound is overlooked when opposing signals cancel each other out.

Hence, chromatographic separation is required to obtain comprehensive and profound information on complex mixtures such as food samples. Current pesticide residue analysis is mostly performed by gas chromatography (GC) or high-performance liquid chromatography (HPLC) with mass spectrometric (MS) detection after dedicated sample preparation [16]. However, coupling of such systems to biological assays, either online or via fraction collection, is inconvenient or even impossible [17, 18]. The organic solvents used for extraction and HPLC separation are incompatible with the bioassay which affords intermediate solvent exchange. Long bioassay incubation times required destroy the previous HPLC separation, making online coupling impossible. Hence, for one sample, fraction collection and solvent exchange are required to perform a subsequent in vitro bioassay and correlate the results of HPLC and bioassay. Of course, such tedious and expensive approaches are not suited for routine sample analysis.

In contrast, a very attractive and sustainable solution to these problems is high-performance thin layer chromatography–effect-directed analysis (HPTLC–EDA) because the organic solvents are evaporated after sample application and chromatography, which eases the bioassay application on the adsorbent layer [17]. The open planar system is ideally suited for the coupling to bioassays and provides image-based effect detection for many samples in parallel [18]. Another advantage is minimal sample preparation, since the technique is highly matrix-tolerant. Hence, the lYES has been transferred to the (HP)TLC field and termed planar YES (pYES), indicating estrogenic activity as MU-blue fluorescent zones (or any other subtrate signals) on the plate. However, the zones on normal phase (NP) HPTLC plates were prone to diffusion [19, 20] for long bioassay incubation times. First in 2014, sharp-bounded zones were obtained on water-wettable reversed-phase (RP-18 W) plates [21, 22] with quantification limits down to 1 pg/zone for 17β-estradiol (E2) and 5 pg/zone for 17α-ethinylestradiol (EE2) [21]. Recently, sharp zones on NP-HPTLC plates with substantial reduction in diffusion were demonstrated via an additional zone fixation step [23]. For further characterization of the active zones, HPTLC–pYES has been coupled to MS [21] and high-resolution MS (HRMS) [22]. In a recent 12 D hyphenation, one active zone after the other was fully automated online-eluted and transferred out of the bioautogram directly to RP-HPLC–diode array detection (DAD)–HRMS/MS [24].

Since the current food analysis using target or non-target LC/GC–MS is unable to detect biological effects [16] and the current in vitro assays (only sum value) obviously fail when opposing signals/effects are present which are not differentiated as it should be [23, 24], it was hypothesized that food safety can be improved and benefit via a non-target HPTLC–pYES screening. In this study, the chromatographic separation and the detection of estrogenic effects were performed on the same surface, which allowed a prioritization on all important (biologically indicated) estrogenic compounds present in a complex sample. Thus, this hyphenation combines and unifies the power and potential of two disciplines (chemistry and biology) on the same surface.

## Materials and methods

### Chemicals and materials

Double-distilled water was produced by a Heraeus Destamat Bi 18 E (Fisher Scientific, Schwerte, Germany). Acetonitrile (> 99.9%), dimethyl sulfoxide (≥ 99.8%), ethanol (≥ 99.9%), *n*-hexane (≥ 98%), toluene (≥ 99.9%), formic acid (> 98%), anhydrous disodium hydrogen phosphate (p.a.), sodium hydroxide (≥ 99%), l-histidine (HPLC grade), and 4-methylumbelliferyl-β-d-galactopyranoside (MUG, for biochemistry) were purchased from Carl Roth (Karlsruhe, Germany). Chloroform (HPLC grade) and diethyl ether (> 99%, stabilized with butylated hydroxytoluene) were obtained from Acros Organics (Thermo Fisher Scientific, Geel, Belgium). Ethyl acetate (≥ 99.8%) and isopropanol (≥ 99.8%) were delivered by Th. Geyer (Renningen, Germany). Copper(II) sulfate pentahydrate, glucose (99.5%), l-adenine (99%), l-aspartic acid (99.5%), l-glutamine (99%), l-glycine (99%), l-isoleucine (99%), l-leucine (99%), l-methionine (98%), l-serine (99%), l-threonine, and yeast nitrogen base without amino acids and ammonium sulfate (for biochemistry) were purchased from Fluka (Sigma-Aldrich, Steinheim, Germany). Methanol (100%) was purchased from VWR (Darmstadt, Germany). l-Lysine hydrogen chloride (analytical grade) was obtained from Serva Feinbiochemica (Heidelberg, Germany). l-Phenylalanine (99%) was delivered by Bachem (Bubendorf, Switzerland). Citric acid monohydrate (99.5–100.5%), l-arginine (for biochemistry), l-tyrosine (for biochemistry), and HPTLC silica gel 60 RP-18 W 20-cm × 10-cm plates were obtained from Merck (Darmstadt, Germany). 17β-Estradiol (E2) was purchased from Dr. Ehrenstorfer (Augsburg, Germany). *Saccharomyces cerevisiae* BJ3503 containing the hERα or hERβ were thankfully obtained from Mcdonnell [13] or Heberle-Bors [25], respectively. Sixty pesticides from five different groups (acaricides, insecticides, fungicides, herbicides, and nematicides) were obtained as listed (Tables S1 and S2). The six PPPs were obtained from various vendors (Table S3). Various tomatoes as well as red and white table grapes from different geographical origins and vendors were obtained from local supermarkets (as specified). One Rivaner white wine sample of 2014 was self-produced (Seiferling, Stettfeld, Germany), and thus, the PPP spray schedule (Table S4) was known.

### Solutions prepared

Each liquid pesticide solution (Table S1, 1 µg/µL each) was 1:200 diluted (5 ng/µL), and further 1:5, 1:10, or 1:50 diluted in the respective solvent to obtain 1, 0.5, or 0.1 ng/µL standard solutions (Table S2). Each solid pesticide was dissolved (1 µg/µL) and further 1:5, 1:50, or 1:500 diluted in the respective solvent to obtain 200, 20, and 2 ng/µL standard solutions (Tables S1 and S2). The E2 (2 mg) was dissolved in 1 mL methanol to obtain a 2-µg/µL stock solution, and a dilution series was prepared using methanol, which resulted in 50 ng/µL, 2.5 ng/µL, 50 pg/µL, and 5 pg/µL concentrations. From the six commercial PPPs (Table S3), the four solid products were dissolved in water (1 µg/µL each), i.e., Dithane^®^ NeoTec (mancozeb), FOLPAN^®^ 80 WDG (folpet), SWITCH^®^ (fludioxonil and cyprodinil), and Teldor^®^ (fenhexamid), whereas the two liquid products, i.e., DYNALI^®^ (difenoconazol and cyflufenamid) and Vivando^®^ (metrafenone), were 1:10 diluted in water. These six stock solutions were 1:10 and 1:50 diluted with methanol, and the resulting concentrations of the active pesticides therein are listed (Table S3). All solutions were stored at − 25 °C in the dark.

### Cell culture prepared

The growth medium was prepared by dissolving 6.8 g yeast nitrogen base without amino acids, 10 g/L d-glucose, and 14 amino acids in different concentrations [26] in 1 L double-distilled water. A cryostock (1 mL) of *Saccharomyces cerevisiae* BJ3503 containing the hERα was diluted in 29 mL of the medium and incubated overnight (18–19 h) at 30 °C at 75 rpm on an orbital shaker (Edmund Bühler, Hechingen, Germany). Polypropylene boxes (KIS 26.5 cm × 16 cm × 10 cm, ABM, Wolframs-Eschenbach, Germany) with filter paper lining were moistened with water at least 30 min before the assay. The cell number was determined out of a 1:10-dilution of the yeast cell suspension in a 0.9% sodium chloride solution using a hemocytometer (Brand, Wertheim, Germany). The cell number was adjusted to 8 × 10^7^ cells/mL by centrifugation (2500 × *g*, 5 min) of the calculated yeast culture volume followed by resuspension of this portion in 40 mL fresh medium plus 200 µL copper sulfate solution (7 µg/µL) [21].

### Sample extraction

The white wine sample (8 mL) was shaken with 5 mL diethyl ether/*n*-hexane 4:1 (*V*/*V*) three times for 30 s in a test tube [24]. After phase separation, the upper phase was evaporated to dryness under nitrogen and redissolved in 100 µL methanol [24]. The skin of grapes and tomatoes was carefully peeled and separated from the pulp. Each skin or pulp sample (1 g) was extracted with 5 mL diethyl ether/*n*-hexane 4:1 (*V*/*V*) by stirring in a 5-mL glass vial for 1 h. The upper phase was evaporated to dryness under nitrogen, and the residue was redissolved in 200 µL methanol and centrifuged at 16,000 × *g* for 5 min. The supernatants were transferred to autosampler vials stored at − 25 °C in the dark.

### Tomato spiking for skin penetration

Cherry tomatoes were washed and halved. One-half was kept as negative control. On the other skin surfaces, 5 µL of either cyprodinil (1 µg/µL) or fenhexamid or fludioxonil (both 0.2 µg/µL each) was evenly applied four times (in total 20 µL spiked on the surface) and incubated at room temperature for 24 h. To simulate a typical household washing process, the halves were rinsed three times with 5 mL water without rubbing the surface. One tomato half spiked with fludioxonil was additionally rubbed with a cotton swab (after the wash-water was dried on the surface). Then the skin was peeled (0.1 g) from the pulp (0.4–0.7 g) and both were separately extracted as mentioned.

### Initial screening and HPTLC–FLD method

All HPTLC instruments were from CAMAG (Muttenz, Switzerland) and controlled by winCATS software (version 1.4.7.2018). The HPTLC silica gel 60 RP-18 W plates were heated at 120 °C for 1 h (TLC Plate Heater III) for hardening the layer binder. Then the plates were pre-washed up to the upper plate edge using first methanol and second ethyl acetate, each followed by plate drying. For the initial screening (without separation), each pesticide standard solution was applied as 7-mm band at different amounts (Table S2, 1–20 µL, mostly 0.002–10 µg/band) with an application speed of 150–250 nL/s depending on the solvent (Automatic TLC Sampler 4 with FreeMode option).

**Fig. 1:**
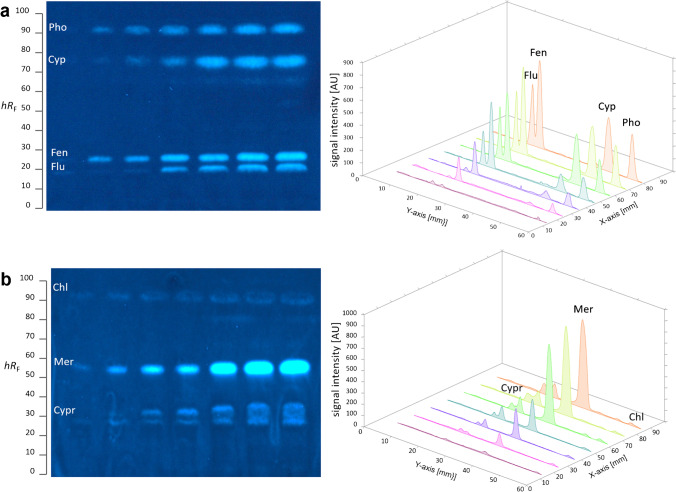
Pesticide screening: HPTLC–pYES–FLD bioautograms at 366 nm of pesticides (Table S1) showing MU-blue fluorescent estrogenic responses and corresponding biodensitograms at 366 nm/ > 400 nm for (**a**) fludioxonil (Flu; 0.06–1 µg/band), fenhexamid (Fen; 0.1–2 µg/band), cypermethrin (Cyp; 3–65 µg/band), phorate (Pho; 1–10 µg/band), (**b**) cyprodinil (Cypr; 3–25 µg/band), mercaptodimethur (Mer; 1–20 µg/band), and chlorpyrifos (Chl; 3–25 µg/band), separated on HPTLC plate silica gel 60 RP-18 W with *n*-hexane/ethyl acetate 5:1 (*V*/*V*) up to 60 mm

The following solutions were applied as 7-mm bands (10-mm distance to the lower plate edge and 15 mm from the side plate edge) with an application speed of 200 nL/s. Individual pesticide standard solutions were oversprayed in increasing amounts as indicated (Fig. 1) and separated with a mixture of *n*-hexane/ethyl acetate 5:1 (*V*/*V*) up to 60 mm (from the lower plate edge) in a Twin-Trough Chamber. The PPP solutions (Table S3, 30 µL) were separated with *n*-hexane/toluene/ethyl acetate 4:1:1 (*V*/*V*/*V*) up to 70 mm, the white wine extract (35 µL) with *n*-hexane/ethyl acetate 5:1 (*V*/*V*) up to 60 mm, and the grape (40 and 60 µL) and tomato extracts (40 µL) with *n*-hexane/toluene/ethyl acetate 5:1:1 (*V*/*V*/*V*) up to 70 mm. Chromatograms were dried in a cold air stream for 4 min and documented via fluorescence light detection (FLD) at 366 nm (automatic exposure, TLC Visualizer 2).

### pYES bioassay application

For neutralization of the RP-18 W plates (per se acidic from the manufacturer’s side) [21], the chromatograms were immersed into a citrate phosphate buffer (citric acid 6 g/L, disodium hydrogen phosphate 10 g/L, adjusted to pH 12 with sodium hydroxide) with an immersion speed of 3.5 cm/s and immersion time of 5 s (TLC Immersion Device), followed by drying in a cold air stream for 4 min. Then the chromatogram was immersed into the prepared yeast cell suspension followed by gentle manual tapping on the glass back of the plate until the wet gloss on the adsorbent surface was gone. It was then incubated horizontally in the humidified polypropylene box (nearly 100% relative humidity) in an oven at 30 °C for 3 h. After drying for 4 min, the chromatogram was immersed into a MUG solution (16 mg MUG in 1 mL dimethyl sulfoxide and 39 mL citrate phosphate buffer) and incubated at 37 °C for 1 h. The dried bioautogram was recorded at FLD 366 nm (automatic exposure, Reprostar 3) for documentation, and optionally, bioautograms were densitometrically scanned at FLD 366 nm/ > 400 nm (TLC Scanner 3).

### Dose–response curves, EC_50_, and E2Eq values of pesticides

On three different days (*n* = 3), sigmoidal seven-point dose–response curves as well as the EC_50_ values (Quest Graph™ IC50 Calculator [27]) were determined for seven pesticides using peak height (peak area for cyprodinil) obtained from the biodensitograms. Therefore, cypermethrin (3–65 µg/band), fenhexamid (0.1–2 µg/band), fludioxonil (0.06–1 µg/band), and phorate (1–10 µg/band) were oversprayed as well as chlorpyrifos (3–25 µg/band), cyprodinil (3–25 µg/band), and mercaptodimethur (1–20 µg/band) and analyzed as described. On each day, also a six-point dose–response curve for E2 (2–20 µL, 5 pg/µL E2 solution, 10–100 pg/band) and the respective EC_50_ and E2Eq (EC_50 E2_/EC_50 sample_) values were calculated.

### Mass spectrometry of prioritized active compound zones

For further characterization of selected estrogenic zones, samples and standards were analyzed twice on the same plate. After plate cut, one plate part was subjected to the pYES bioassay, the other was used for MS measurement. The active zones in the bioautogram were marked on the MS plate using a soft pencil. The zones were online transferred with methanol at a 0.1-mL/min flow rate using the Plate Express (Advion, Ithaca, NY, USA) and an HPLC pump (MX010PFT, Teledyne SSI, State College, PA, USA) into a single quadrupole MS (Expression CMS, Advion, Ithaca, NY, USA) equipped with an electrospray ionization (ESI) source. Full scan mass spectra were recorded in the positive ionization mode with the following settings: capillary temperature 250 °C, capillary voltage 100 V, source voltage offset 25 V, source voltage span 25, source gas temperature 250 °C, and ESI voltage 3.5 kV.

## Results and discussion

The xenoestrogenic activity of 60 pesticides, six PPPs, and three different food sample types (tomato, grape, and wine) was investigated for the first time using a non-target planar estrogenic screening technique. Dose–response curves as well as half-maximal effective concentrations (EC_50_) and E2 equivalents (E2Eq values) were exemplarily determined for seven estrogenic pesticides. Increasing in sample complexity, the PPPs and food samples were studied for estrogenic acting compounds and for opposing effects that generally impairs the estrogenic signal detection in current in vitro microtiter plate assays.

### Screening of 60 pesticides for xenoestrogenic activity

Xenoestrogenic activity was reported for various pesticides in several in vitro [4, 5, 7–9] and in vivo [4, 5, 28, 29] assay studies. For the first time, the planar assay on the adsorbent surface was studied for screening of the xenoestrogenic activity of 60 arbitrarily selected pesticides (Table S1). For a rapid overview on potential responses, chromatography was skipped, and each of the 60 pesticide compounds was merely applied at different amounts in a wide concentration range (Table S2, mostly 0.002–10µg/band) on the HPTLC plate silica gel 60 RP-18 W. After the pYES bioassay application, the estrogenic activity was indicated as blue fluorescent MU band, produced from MUG by the released galactosidase upon receptor binding of an estrogenic compound. Ten out of 60 pesticides, namely carbaryl, chlorpyrifos, cypermethrin, cyprodinil, fenhexamid, fludioxonil, mercaptodimethur, pendimethalin, phorate, and picoxystrobin, exhibited prominent ERα-mediated estrogenic effects (Fig. S1a). Using the hERβ receptor instead (use of the other cell strain is the only difference in the workflow), fenhexamid also showed a response though weaker (Fig. S1b). Five of these, i.e., cyprodinil, cypermethrin, fenhexamid, fludioxonil, and pendimethalin, are currently approved in the EU [3].

**Fig. 2:**
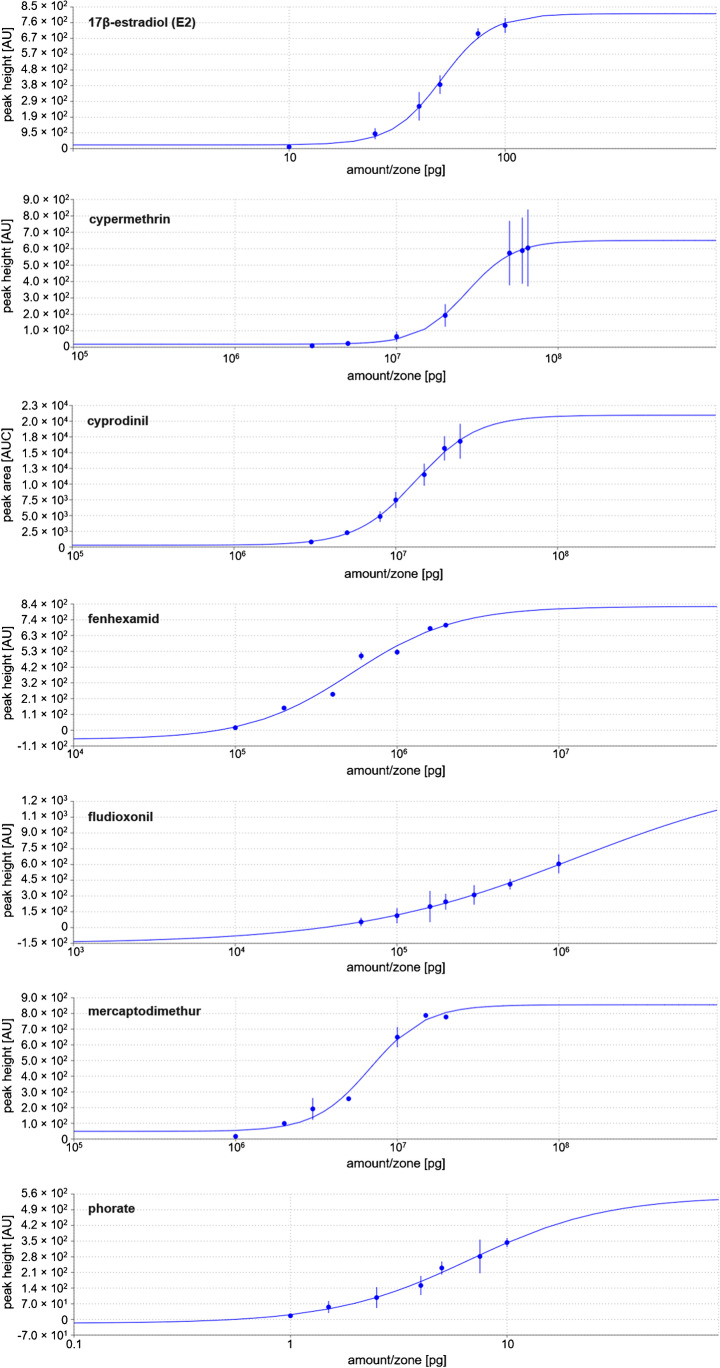
Dose–response curves (Table S2) exemplarily determined for the estrogenic responses of seven pesticides obtained from biodensitograms on three different days (*n* = 3; standard deviation as error bar)

Carbaryl, pendimethalin, and picoxystrobin were only active in the initial experiments (without separation) but this was not confirmed in the following HPTLC–pYES experiments, which was explained by instability of the standard solutions but requires further detailed studies. For the seven other pesticides, reproducible estrogenic effects were observed as MU-blue fluorescent bands (Fig. 1). After this initial screening, dose–response curves were determined for the seven active pesticides. The concentration range was chosen for each of those individually, ensuring good detectability without overloading the plate. Each pesticide standard solution was applied in seven ascending amounts per band in an overspray mode to generate a mixture of four (fludioxonil, fenhexamid, cypermethrin, and phorate, Fig. 1a) or three (chlorpyrifos, cypermethrin, and mercaptodimethur, Fig. 1b) pesticides, which were separated using *n*-hexane/ethyl acetate 5:1. The estrogenic signal responses of the pesticides obtained from biodensitometric measurement (Fig. 1) of the bioautograms repeated on three different days were used to determine sigmoidal dose–response curves [30] for E2 as well as the pesticides cypermethrin, cyprodinil, fenhexamid, fludioxonil, mercaptodimethur, and phorate (Fig. 2). No dose–response dependency was observed for chlorpyrifos. From the dose–response curves, EC_50_ values were determined for E2 and the six pesticides, from which E2Eq values were calculated to evaluate the ERα receptor affinity (Table 1). Based on the E2Eq values, the hERα receptor affinity of the investigated pesticides can be assessed as low, and the affinity decreased in the order fenhexamid > fludioxonil > phorate > mercaptodimethur > cyprodinil > cypermethrin (Table 1).

**Table 1:** EC_50_ and E2Eq values determined for the E2-reference and the pesticides

Compound	EC_50_ (pg/band)	E2Eq
E2	51	1.0
Cypermethrin	2.7 × 10^7^	1.9 × 10^−6^
Cyprodinil	7.7 × 10^6^	4.2 × 10^−6^
Fenhexamid	5.3 × 10^5^	9.6 × 10^−5^
Fludioxonil	1.3 × 10^6^	4.0 × 10^−5^
Mercaptodimethur	6.8 × 10^6^	7.5 × 10^−6^
Phorate	6.7 × 10^6^	7.6 × 10^−6^

To the best of our knowledge, xenoestrogenic activity was described in the literature for all pesticides that were tested positive in the pYES, except for phorate, which should be clarified by further studies. Chlorpyrifos [31], cyprodinil [32], fludioxonil [31, 32], and fenhexamid [31, 32] exhibited estrogenic effects in the lYES assay. For chlorpyrifos, a dose-dependent effect was observed in the lYES [31] and other assays [33], whereas in the current pYES study, an estrogenic response but no significant dose-response dependency was observed in the studied range of 0.02–10µg/band, which therefore requires further investigations. For cypermethrin, no lYES study is available, but it has been shown to activate hERα in the E-screen assay [34] and human BG-1 ovarian adenocarcinoma cells [35]. Mercaptodimethur, carbaryl, and some of their metabolites exhibited estrogenic activity in the E-screen assay [36] and other cell lines [8, 36]. No ERα-mediated activity was found for phorate in Chinese hamster ovary (CHO) cells [8], whereas a dose-dependent effect was observed in the current study, which should be clarified in future studies. Pendimethalin and picoxystrobin, which only exhibited estrogenic effects in our very first experiments, have also been demonstrated to possess estrogenic potential. For pendimethalin, both estrogenic and antiandrogenic activity in the E-screen [37] and CHO cells [8] has been reported and picoxystrobin showed estrogenic activity in multiple assays [33].

Although the receptor affinities (E2Eq values) of the pesticides that were tested positive in the pYES study were low compared to E2, several pesticides are commonly used during food cultivation (spray schedule) and commercial PPPs often consist of several pesticides as active ingredients (Table S3). This gives rise to accumulation and mixture effects even at very low doses [10, 31] through additive or synergistic action [23], making their low-level detection very important, even if the receptor affinity is poor. Recently, it was observed in planar bioautograms using the planar yeast antagonist estrogen screen (pYAES) bioassay that most out of the 68 plant-based extracts studied showed a synergistic endocrine effect [23, 38]. The receptor binding of the agonist stripe applied along each separated sample track was increased by non-active compounds in the plant-based extracts. Since pesticides are applied on fruits and vegetables, such synergistic effects need detailed research using the pYAES bioassay as an ideal tool. In real-world scenarios, therefore, the impact on hERα activation can be easily underestimated by considering only a low affinity of a single pesticide. It must also be considered that in this exemplarily study, only hERα-mediated estrogenic effects were investigated, although pesticides can activate the hERβ as well in an additive manner [31]. In 2019, 2.3% of all samples analyzed in the EU were found to be non-compliant, meaning pesticide residues exceeded the MRLs, while a very high percentage of 40% of compliant food samples contained quantifiable residues but below the regulatory limits [39], which can still be problematic due to the mentioned accumulation and mixture effects.

### Xenoestrogenic activity of six commercial PPPs

**Fig. 3:**
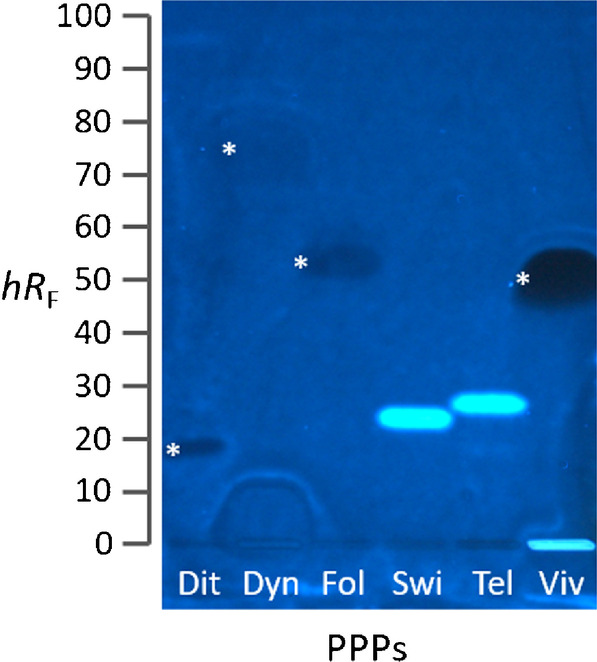
PPP screening: HPTLC–pYES–FLD bioautogram image at 366 nm of six commercial PPPs (Table S3) showing blue fluorescent estrogenic responses but also opposing effects (dark bands marked with asterisk indicate true anti-estrogens or false-positive anti-estrogens or cyctotoxins) on HPTLC plate silica gel 60 RP-18 W, developed with *n*-hexane/toluene/ethyl acetate 4:1:1 (*V*/*V*/*V*) up to 70 mm. Dit, Dithane^®^ NeoTec (mancozeb, 2.3 µg/band); Dyn, DYNALI^®^ (difenoconazol and cyflufenamid, 0.2 and 0.9 µg/band, respectively); Fol, Folpan^®^ 80 WDG (folpet, 2.4 µg/band); Swi, SWITCH^®^ (fludioxonil and cyprodinil, 0.8 and 1.1 µg/band, respectively); Tel, Teldor^®^ (fenhexamid, 1.5 µg/band); Viv, Vivando^®^ (metrafenone, 1.5 µg/band)

Increasing in sample complexity, the potential of the highly efficient and powerful effect differentiating planar estrogenic screening technique was studied next. Six PPPs (Table S3) were investigated to prove the applicability of HPTLC–pYES for the detection of xenoestrogens in commercial ready-to-use formulations. After an initial screening (Fig. S2), the diluted PPP solutions were applied on an RP-18 W plate and separated with *n*-hexane/toluene/ethyl acetate 4:1:1. As expected, estrogenic effects were observed for the products SWITCH^®^ (Fig. 3, *hR*_F_ 24), containing fludioxonil and cyprodinil, and Teldor^®^ (*hR*_F_ 27), containing fenhexamid. Although SWITCH^®^ consists of two xenostrogenic pesticides, only one fluorescent band was visible in this PPP. This was not surprising, as the EC_50_ determined for cyprodinil was 7.7 µg/band (Table 1), but the applied cyprodinil amount (contained in the PPP) was 1.1 µg/band (30 µL of the 1:10 diluted PPP; Table S3), and thus only fludioxonil (0.8 µg/band; Table S3) was visible whose EC_50_ was 1.3 µg/band (Table 1). DYNALI^®^, which contained cyflufenamid and difenoconazol, showed a weak estrogenic effect, evident as blue halo fluorescence around the lower one of these two pesticides. Note that such halo-effects can be proven using piezoelectric spraying of the bioassay suspensions [24]. Vivando^®^, which contains metrafenone, revealed a native fluorescent zone in the application area and a dark, wide-spread zone at hR_F_ 52. In contrast to common in vitro assays, i.e., lYES, in which opposing signals/effects falsify the estrogenic signal response detection as a sum value, here the opposing dark zones were clearly separated from the estrogenic signal and differentiated. Dark zones were also observed for Dithane^®^ NeoTec (hR_F_ 18), DYNALI^®^ (hR_F_ 75), and Folpan^®^ (hR_F_ 54). Dark zones indicate either true anti-estrogenic, false-positive anti-estrogenic, or cytotoxic effects. This can be clarified by differentiation of the individual effects in the performance mode as a multiplex bioassay, termed planar yeast antagonist verified estrogen screen (pYAVES) bioassay [23, 38]. Briefly, before the bioassay application, two stripes (i.e., of the E2 agonist and MU end-product) were applied along each separated sample track, and the so prepared chromatogram was subjected to the bioassay. These stripes help to clarify the mechanism of the dark zones in the bioautogram. A true antagonist would interrupt the E2 stripe by blocking the hERα for E2 (biological response) but not the MU stripe. In contrast, a false-positive antagonist would also reduce the MU fluorescence (physico-chemical fluorescence reduction). In this way, it is also possible to detect synergistic effects as a comparatively more intense blue fluorescence at a position where the E2 stripe overlaps a band that is otherwise not active. An integrated detection with a tetrazolium salt substrate or resazurin substrate can detect cytotoxicity [23]. Hence, this dark zone formation of some PPPs should be investigated using such multiplex bioassay performance in the future.

### (Xeno)estrogenic pesticide residues in food samples

A unique powerful benefit of the non-target HPTLC–EDA technique is the combination of chromatographic separation and effect detection on the same surface [18]. The detection is non-target and points to all important compounds exhibiting a certain effect, including analytes outside the analytical focus, such as isomers, degradation products, metabolites, and other biotransformation products, which are considered the *blind spot* [7] of pesticide residue risk assessment. Due to the chromatographic separation, the observed effects can be directly linked to the responsible compound zone. Thus, HPTLC–EDA is ideally suited to screen complex food samples for (xeno)estrogenic compounds. Hence, tomatoes, grapes, and a white wine sample for which the PPP spray schedule (Table S4) was known were investigated via HPTLC–pYES.

**Fig. 4:**
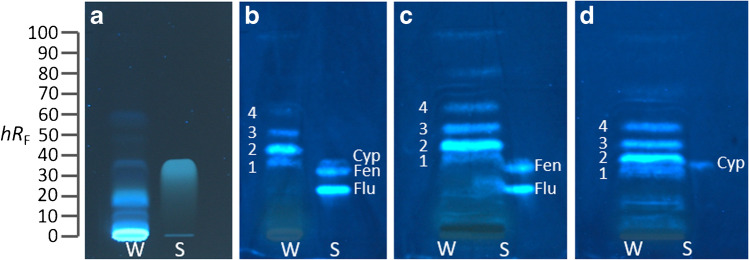
White wine screening: (**a**) HPTLC–FLD chromatogram at 366 nm and (**b**–**d**) corresponding HPTLC–pYES–FLD bioautograms of the Rivaner white wine sample (W, 35 µL/band) showing the blue fluorescent estrogenic zones **1**–**4** (only slight opposing effects evident) in comparison to the pesticides (S) fludioxonil (Flu; 1.2 µg/band), fenhexamid (Fen; 1 µg/band), and cyprodinil (Cyp; 30 µg/band) as (**b**) separate tracks or (**c**, **d**) overlapped with the sample, which revealed (**c**) fenhexamid residues in zone **1** and possibly also (**d**) cyprodinil, separated on HPTLC plate silica gel 60 RP-18 W with *n*-hexane/ethyl acetate 5:1 (*V*/*V*) up to 60 mm

The white wine sample which was extracted with diethyl ether/*n*-hexane 4:1 [24] and separated with *n*-hexane/ethyl acetate 5:1 revealed the MU-blue fluorescent estrogenic zones **1**–**4** (Fig. 4b–d). The sample was compared to the pesticides fludioxonil, fenhexamid, and cyprodinil, all used during grape cultivation (Table S4). Zone **1** was suspected to be caused by either fenhexamid or cyprodinil. Both pesticides were applied overlapped with the wine sample and separated (Fig. 4c, d) to prove this assumption. This showed that residues of fenhexamid were present in the wine sample, since the estrogenic zone **1** overlapped with the fenhexamid band (Fig. 4c), but at slightly different *hR*_F_ values caused by the wine matrix. The presence of the other pesticide cyprodinil could not be unequivocally clarified because the overlapped cyprodinil band was partially hidden by the bright fluorescent zone **2**. Hence, zones **2**–**4** remained unknown but could have been caused by (photo)degradation/oxidation/hydrolysis or by phytoestrogens [24]. Considering that the estrogenic responses were obtained from 8 mL wine extracted in 100 µL, of which 35 µL was applied, drinking a 200-mL glass of wine would contribute with the 70-fold estrogenic effect than observed in the bioautogram (Fig. 4). Further studies are needed to clarify how this estrogenic effect affects consumers when they drink wine, aside from ethanol intake.

**Fig. 5:**
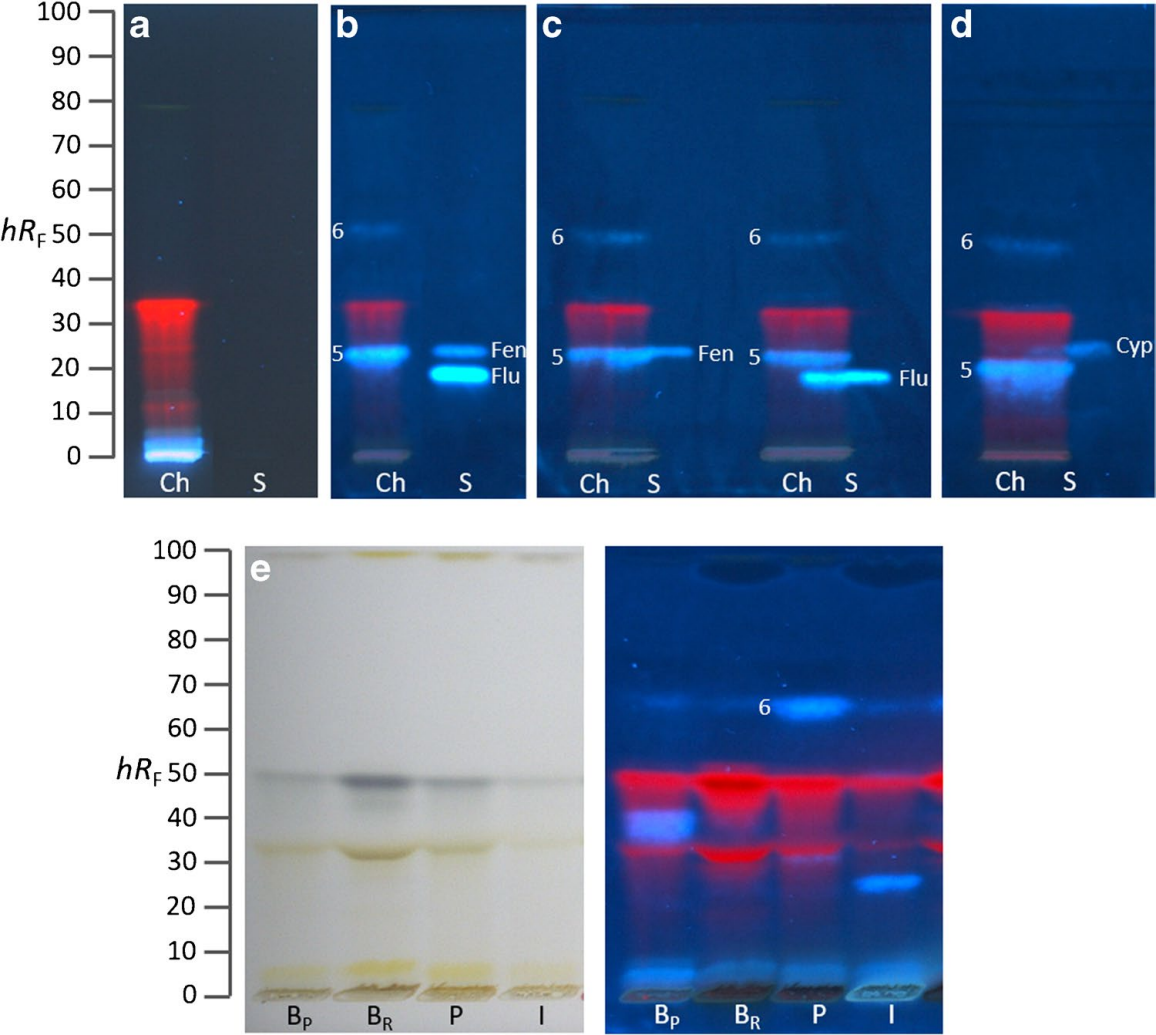
White seedless table grape skin screening: (**a**) HPTLC–FLD chromatogram at 366 nm and (**b-d**) corresponding HPTLC–pYES–FLD bioautograms of the skin of a grape from Chile (Ch, 40 µL/band) showing blue fluorescent estrogenic zones **5** and** 6**, which revealed fenhexamid residues in zone **5**, whereas zone **6** was present in all 18 grape samples studied, in comparison to the pesticides (S) fludioxonil (Flu; 1.2 µg/band), fenhexamid (Fen; 1 µg/band), and cyprodinil (Cyp; 30 µg/band) as (**b**) separate track or (**c**, **d**) overlapped with the sample, separated on HPTLC plate silica gel 60 RP-18 W with *n*-hexane/toluene/ethyl acetate 5:1:1 (*V*/*V*/*V*) up to 70 mm. (**e**) Further screening (with higher elution power using *n*-hexane/toluene/ethyl acetate 4:1:1, *V*/*V*/*V*) of samples from Brazil (B, Festival Seedless bought from Penny_P_ and Sugar Crispy from REWE_R_), Peru (P, Prime Seedless from Lidl), and Italy (I, from local market), detected at white light illumination and FLD 366 nm, all 60 µL/band

The extractant diethyl ether/*n*-hexane 4:1 [24] used for the wine sample was also applicable for grapes and tomatoes. The mobile phase composition was adjusted to *n*-hexane/toluene/ethyl acetate 5:1:1. Different tomato and white table grape samples from different geographical origins and vendors were screened for estrogenic activity via HPTLC–pYES. No estrogenic activity was found in the pulp of the grapes. In the grape skin samples, estrogenic zone **5** was detected (Fig. 5b–d), exemplarily shown for the grapes from Chile. Using overlapping application of pesticide standards, zone **5** was shown to be fenhexamid (Fig. 5c). Zone **6** was present in the skin of all table grape samples (all in all 18 different grape samples were tested), suggesting a phytoestrogen. In addition, red fluorescent chlorophylls were separately detected as well as the already discussed dark zones (opposing signals/effects), which falsify the sum signal response detection in in vitro assays.

**Fig. 6:**
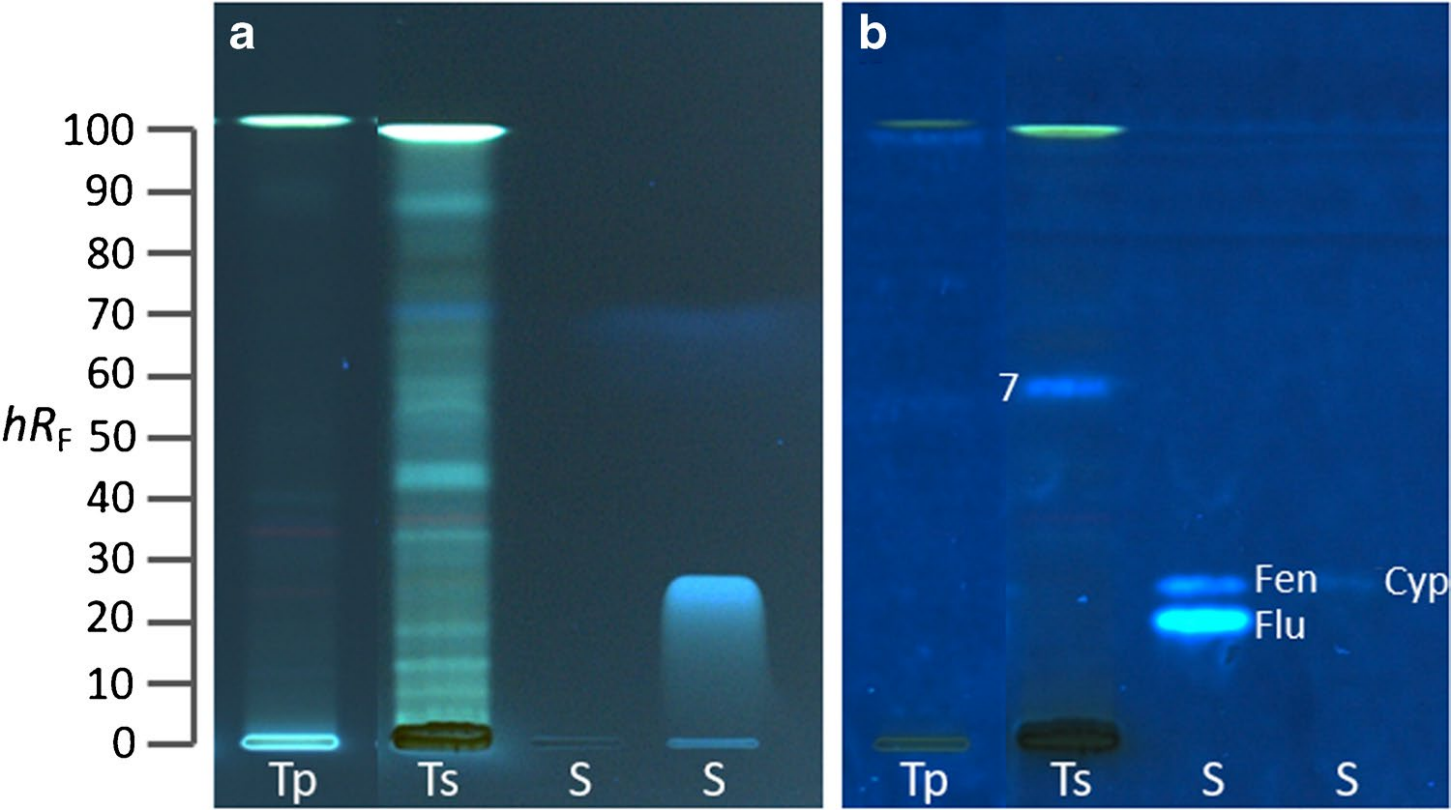
Tomato screening: (**a**) HPTLC–FLD chromatogram at 366 nm and (**b**) corresponding HPTLC–pYES–FLD bioautogram of pulp (tp) and skin (ts) of a cherry tomato from Belgium (40 µL/band, bought from Penny) showing the blue fluorescent estrogenic zone **7** present in all samples, in comparison to the pesticides (S) fludioxonil (Flu; 1.2 µg/band), fenhexamid (Fen; 1 µg/band), and cyprodinil (Cyp; 30 µg/band) separated as in Fig. 5

In the studied tomato samples (all in all ten different samples were tested), no response was observed in the *hR*_F_ region of the pesticide standards (Fig. 6). In all 10 different tomato skins, zone **7** was detected (Fig. 6b), which can indicate a phytoestrogen but is more likely a reaction product of cyprodinil with the tomato skin (as discussed in the following section). Again, in tomato skin samples, a strong dark zone was observed at the application zone. Here, the opposing response is clearly separated from the estrogenic responses, which, however, is not the case for sum values of in vitro assays. Considering that the estrogenic responses were obtained from 1 g grapes or tomatoes extracted in 200 µL, of which 40 µL was applied, eating 200 g grapes or tomatoes would contribute with the 1000-fold signal effect than observed in the bioautogram (Figs. 5 and 6), which effect on the health of consumers should be clarified.

Based on the examples described, the potential of HPTLC–pYES for screening for xenoestrogenic pesticides and other estrogenic compounds in food has been demonstrated. The unknown zones should be further characterized using either direct coupling to HRMS or even more advanced multi-hyphenations [24], which was not available at the time of the study. Also the latest zone fixation on NP-HPTLC plates [23] could be studied to improve the limit of detection. Although we studied several extractants, other extraction solvents could be tested to ensure that all extractable estrogens are considered or to increase the range of extractable estrogenic compounds. As mentioned, effect differentiation is the key [40], which was recently discovered for genotoxic compounds found in plant-based oils used and recommended in healthy diets. We will never make progress in understanding when the sum values are wrong in case of complex samples.

### Pesticide penetration through tomato skin

**Fig. 7:**
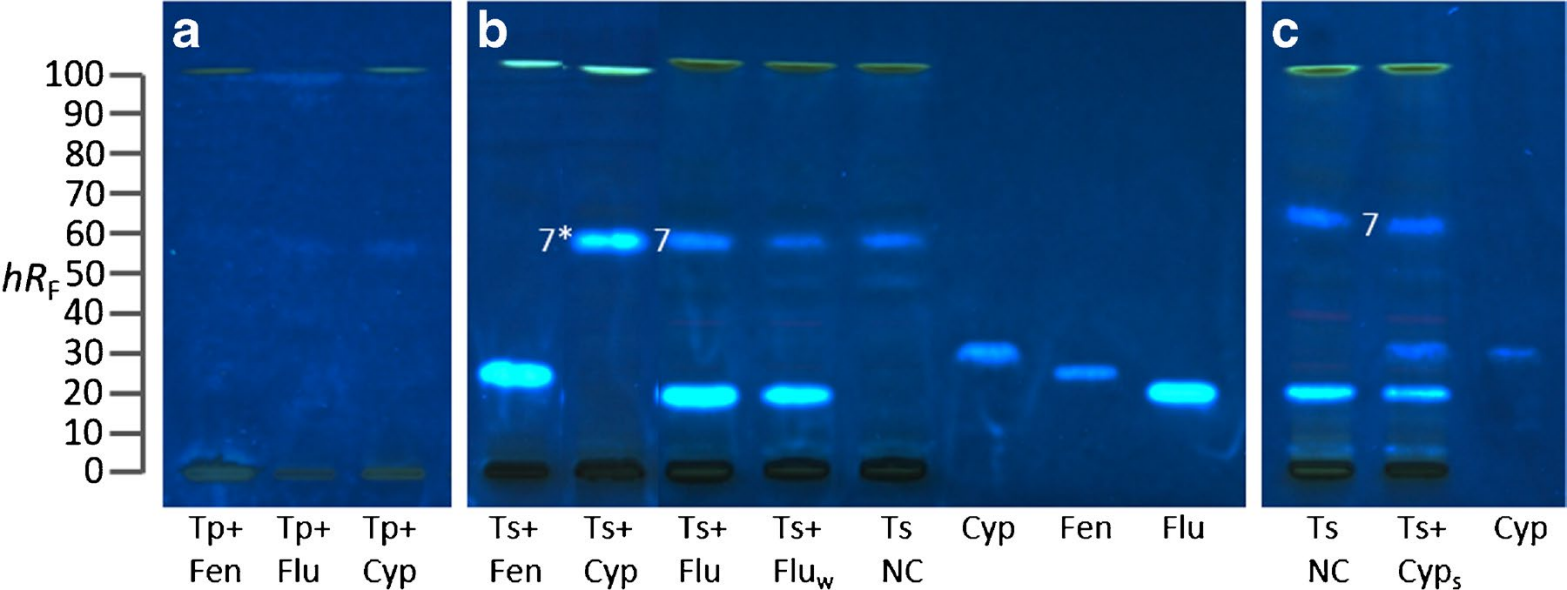
Household tomato washing experiment: (**a**) HPTLC–pYES–FLD bioautograms at 366 nm of tomato pulp (Tp, 40 µL/band) or (**b**) tomato skin (Ts, 40 µL/band, same tomato sample as in Fig. 6) treated either with fludioxonil (Flu; 4 µg/halved tomato), fenhexamid (Fen; 4 µg/halved tomato), or cyprodinil (Cyp; 20 µg/halved tomato) (**a**, **b**) before extraction, or (**c**) spiked (_s_) post-extraction and (**b**) skin wiped with a cotton swab (_W_) in comparison to negative control (NC) and pesticide standards, separated as in Fig. 5. Zone **7** increased in the response by the treatment with cyprodinil (7*)

For the reduction of pesticide residues in fresh fruits and vegetables, they are often washed with water before consumption or further processing. Depending on the type of food and the physicochemical properties of the pesticide, they are hardly to completely removed by washing [41]. Pesticides can also irreversibly be bound to the cuticle [42], for which the washing does not help. To exemplarily investigate the behavior of some xenoestrogenic pesticides, fludioxonil, fenhexamid, and cyprodinil were applied onto the surface of cherry tomato halves, and these were incubated for 24 h. The halves were then rinsed with double-distilled water and one-half containing fludioxonil was additionally wiped with a clean cotton swab to simulate rubbing performed during vegetable washing in a common household. The skin was carefully peeled from the pulp and both were extracted and investigated via HPTLC–pYES–FLD. In the pulp (Fig. 7a), no residues of the pesticides that were applied on the surface were found, indicating that the pesticides were not able to penetrate through the tomato skin during the 24-h period. In the skin (Fig. 7b), the residues of fenhexamid and fludioxonil were found at the same *hR*_F_ as the standard compounds; thus, they were not removed by washing with water. The cherry tomato half that had been wiped with the cotton swab showed only a very slightly reduced fluorescence, demonstrating that rubbing does not contribute much to pesticide reduction while washing. The half that had been treated with cyprodinil, however, did not show a fluorescent band at the expected same position as the cyprodinil standard but enhanced the fluorescence of zone 7*. This indicates a reaction product of cyprodinil with the tomato skin or a degradation product. Since this zone **7** was abundant in the ten tomato sample skins studied, the exact mechanism of formation of this cyprodinil derivative should be clarified in the future. As a further test, the negative tomato control sample was spiked with cyprodinil post-extraction to let cyprodinil react with the tomato skin extract. In contrast to the simulated 24-h tomato skin contact, this short contact period did not enhance zone **7**, and the spiked cyprodinil was positioned at the same *hR*_F_ as the cyprodinil standard (Fig. 7c). To prove the presence of a degradation/reaction product upon longer contact with the tomato skin, an identical plate was prepared but without bioassay application, and the zones of interest were subjected to HPTLC–MS (Fig. S3). As expected, the cyprodinil standard zone showed a mass signal at *m*/*z* 226, which corresponded to the protonated molecule. The base peak of the mass spectrum of zone **7** revealed a base peak at *m*/*z* 433, which was also present in the enhanced zone 7*. In the latter, an additional peak at *m*/*z* 239 was detected with almost the same intensity as the base peak, which could be a biotransformation product of cyprodinil. HRMS systems or advanced multi-hyphenations [24] were not available at the time of the study to obtain molecular formulas of the unknown zones.

It showed that rinsing with water is not sufficient to remove residues of either fludioxonil, fenhexamid, or cyprodinil. It illustrated that, though not usually performed for tomatoes, peeling would be more appropriate, as it is very effective at removing pesticides remaining on the surface or in the skin of the food [41]. In particular, the use of xenoestrogenic pesticides should be reconsidered, since mixture effects can be harmful to human health and disturb normal human reproductive processes even at low doses [10, 31].

## Conclusions

Current food safety based on in vitro assays (only sum value) and LC/GC–MS techniques (no biological effect detection) should be complemented by planar assay screening. Our very first HPTLC–pYES–FLD experiments in this field already showed the huge potential. The non-target prioritization on the important estrogenic compounds is straightforward thanks to the combination of two disciplines on the same surface, i.e., chromatographic separation and non-target effect detection. A wide variety of 60 pesticides, six PPPs, and three different food types were screened for estrogenic activity. Ten of the screened 60 pesticides exhibited a xenoestrogenic effect. Dose–response curves, EC_50_ values, and E2Eq values of selected pesticides successfully proved the validity of the method. Analysis of the more complex commercial ready-to-use PPP formulations confirmed these results and proved the applicability of the non-target planar assay screening. Applied to the even more complex food samples, the method revealed therein the presence of pesticides previously shown to act as xenoestrogens. Apart from these detected estrogenic compounds, reaction or breakdown products that are estrogenic were also detected, although not in the previous focus. Experiments showed that some pesticides might undergo degradation upon penetration into the skin of the food, and also such active degradation products were detected via the non-target HPTLC–pYES method but would have been missed by target analysis which is commonly used for food safety.

### Supplementary information

Below is the link to the electronic supplementary material.

Supplementary file1 (PDF 411 KB)
