# Supplementary material for: Non-target estrogenic screening of 60 pesticides, six plant protection products, and tomato, grape, and wine samples by planar chromatography combined with the planar yeast estrogen screen bioassay
Source: Anal Bioanal Chem. 2023 Mar 6;416(3):701–13. doi: 10.1007/s00216-023-04605-x (PMC10766744; doi:10.1007/s00216-023-04605-x)
Supplement: Supplementary file 1 — Supplementary file1 (PDF 411 KB) [file 216_2023_4605_MOESM1_ESM.pdf]

**Supplementary Information**

# Non-target estrogenic screening of 60 pesticides, six plant protection products, and tomato, grape and wine samples by planar chromatography combined with the planar yeast estrogen screen bioassay

Annabel Mehl, Sophia Seiferling, and Gertrud E. Morlock*

Institute of Nutritional Science, Chair of Food Science, and Interdisciplinary Research Center (iFZ), Justus Liebig University Giessen, Heinrich-Buff-Ring 26-32, 35392 Giessen, Germany

*Corresponding Author: phone +49-641-99-39141; gertrud.morlock@uni-giessen.de

**Table of Contents**


Page S-3 **Table S1.** List of 60 pesticide standards and 1-µg/µL solutions prepared

Page S-5 **Table S2.** Pesticide concentration range investigated for estrogenic activity

Page S-6 **Table S3.** List of 6 commercial PPPs and solutions prepared

Page S-7 **Table S4.** PPP spray schedule used for the Rivaner white wine 2014 (Seiferling, Stettfeld, Germany)

Page S-8 **Fig S1.** Planar estrogen screening of 10 pesticides applied at different amounts on the HPTLC plate silica gel 60 RP-18 W (without chromatography) showing (**a**) plate images at FLD 366 nm with estrogenic pesticide zones as MU-blue fluorescent bands. (**b**) Comparison of the responses of fenhexamid (100-600 ng/band) using the hERα receptor *versus* hERβ receptor, which shows that the latter has a weaker response.

Page S-9 **Fig. S2.** Planar estrogen screening of 6 PPPs (without chromatography) showing the plate image at FLD 366 nm with estrogen-like PPP zones as MU-blue fluorescent bands.

Page S-10 **Fig S3.** Mass spectra obtained via HPTLC–pYES–MS of the cyprodinil standard (**a**), zone **7** in tomato skin as negative control (**b**), and tomato skin extract treated with cyprodinil (**c**) containing the enhanced unknown zone **7***.

**Table S1.** List of 60 pesticide standards and 1-µg/µL solutions prepared

| ID | Pesticide standard | Class | Manufacturer | Purity [%] | Solvent |
|---|---|---|---|---|---|
| 1 | Acetamiprid (ace) | Acaricide, insecticide | HPC Standards (Cunnersdorf, Germany) | 99.9 | Acetone |
| 2 | Acephate (aceph) | Insecticide | Dr. Ehrenstorfer (Augsburg, Germany) | 99 | Ethanol |
| 3 | Aldicarb (aldi) | Acaricide, insecticide, nematicide | Honeywell Riedel-de Haën (Seelze, Germany) | 99 | Acetone |
| 4 | Amitraz (ami) | Acaricide, insecticide | Dr. Ehrenstorfer (Augsburg, Germany) | 96 | Acetone |
| 5 | Atrazine (atra) | Herbicide | Institute of Organic Industrial Chemistry (Warsaw, Poland) | ≥98.7 | Ethyl acetate |
| 6 | Azoxystrobin (azo) | Fungicide | Fluka, Sigma-Aldrich (Steinheim, Germany) | 99.9 | Acetone |
| 7 | Bentazon (ben) | Herbicide | Dr. Ehrenstorfer (Augsburg, Germany) | 99 | Acetone |
| 8 | Carbendazim (car) | Fungicide | Dr. Ehrenstorfer (Augsburg, Germany) | 98.5 | Acetone/ dimethyl sulfoxide 2:1 (*V*/*V*) |
| 9 | Carbaryl (carb) | Insecticide | Honeywell Riedel-de Haën (Seelze, Germany) | 99 | Isopropanol |
| 10 | Chlorpyrifos (chlo) | Acaricide, insecticide | Dr. Ehrenstorfer (Augsburg, Germany) | 98.4 | Acetone |
| 11 | Chlorpropham (chlor) | Herbicide | n. s. | 99 | Acetone |
| 12 | Cyfluthrin (cyflu) | Acaricide, insecticide | Dr. Ehrenstorfer (Augsburg, Germany) | 94.5 | Isopropanol |
| 13 | Cyprodinil (cyp) | Fungicide | Dr. Ehrenstorfer (Augsburg, Germany) | 97.5 | Acetone |
| 14 | Cypermethrin (cyper) | Acaricide, insecticide | Institute of Organic Industrial Chemistry (Warsaw, Poland) | 98.2 ± 0.2 | Methanol |
| 15 | Dazomet (daz) | Fungicide, herbicide, nematicide | Honeywell Riedel-de Haën (Seelze, Germany) | 99 | Acetone |
| 16 | Desmedipham (des) | Herbicide | Honeywell Riedel-de Haën (Seelze, Germany) | 99 | Acetone |
| 17 | Difenoconazole (dife) | Fungicide | Dr. Ehrenstorfer (Augsburg, Germany) | 99.5 | Acetone |
| 18 | Diflubenzuron (diflu) | Insecticide | Institute of Organic Industrial Chemistry (Warsaw, Poland) | ≥99.7 | Acetone |
| 19 | Dimethomorph (dim) | Fungicide | Dr. Ehrenstorfer (Augsburg, Germany) | 99 | Acetone |
| 20 | Dithianon (dit) | Fungicide | n. s. | 99 | Acetone |
| 21 | Epoxiconazol (epo) | Fungicide | Honeywell Riedel-de Haën (Seelze, Germany) | 99.2 | Acetone |
| 22 | Fe(III)-dimethyldithio carbamate (FeDi) | n. s. | UCB Chemical Sector (Brussels, Belgium) | 98.6 | n. s. |
| 23 | Fenhexamid (fen) | Fungicide | Bayer Cropscience (Monheim am Rhein, Germany) | 99.1 | Isopropanol |
| 24 | Fenpropidin[a] (fenp) | Fungicide | Honeywell Riedel-de Haën (Seelze, Germany) | 97.5 | Acetone |
| 25 | Fenpropimorph[a] (fe) | Fungicide | Fluka, Sigma-Aldrich (Steinheim, Germany) | 93.3 | Methanol |
| 26 | Fludioxonil (flu) | Fungicide | Dr. Ehrenstorfer (Augsburg, Germany) | 99.5 | Ethyl acetate |
| 27 | Folpet (fol) | Fungicide | n. s. | 99 | Isopropanol |
| 28 | Glyphosat (gly) | Herbicide | n. s. | n. s. | Water |
| 29 | Hexythiazox (hex) | Acaricide, insecticide | Dr. Ehrenstorfer (Augsburg, Germany) | 99.4 | Methanol |
| 30 | 2-Imidazolidinone (imi) | n. s. | Honeywell Riedel-de Haën (Seelze, Germany) | 99.9 | Dimethyl sulfoxide/ |

| ID | Pesticide standard | Class | Manufacturer | Purity [%] | Solvent |
|---|---|---|---|---|---|
| | | | | | methanol 1:9 (*V*/*V*) |
| 31 | Iprovalicarb[a] (ipro) | Fungicide | Dr. Ehrenstorfer (Augsburg, Germany) | n. s. | Ethyl acetate |
| 32 | Isoproturon (iso) | Herbicide | Dr. Ehrenstorfer (Augsburg, Germany) | 99 | Methanol |
| 33 | Malathion[a] (mal) | Acaricide, insecticide | Dr. Ehrenstorfer (Augsburg, Germany) | 99.5 | Ethanol |
| 34 | Mancozeb (man) | Fungicide | HPC Standards (Cunnersdorf, Germany) | 99.3 | Dimethyl sulfoxide/ methanol 4:1 (*V*/*V*) |
| 35 | Maneb (mane) | Fungicide | Honeywell Riedel-de Haën (Seelze, Germany) | 90.4 | Chloroform/ dimethyl sulfoxide 1:5 (*V*/*V*) |
| 36 | MCPA | Herbicide | n. s. | 99 | Ethanol |
| 37 | Mecoprop/MCPP | Herbicide | n. s. | 99.2 | Ethanol |
| 38 | Mercaptodimethur (mer) | Insecticide | Dr. Ehrenstorfer (Augsburg, Germany) | 98.5 | Isopropanol |
| 39 | Metalaxyl (metala) | Fungicide | Dr. Ehrenstorfer (Augsburg, Germany) | 99.5 | Ethanol |
| 40 | Metamitron (meta) | Herbicide | Dr. Ehrenstorfer (Augsburg, Germany) | 99 | Ethyl acetate |
| 41 | Metconazole (metco) | Fungicide | Dr. Ehrenstorfer (Augsburg, Germany) | 99.0 | Methanol |
| 42 | Metiram (met) | Fungicide | Dr. Ehrenstorfer (Augsburg, Germany) | 42 | Dimethyl sulfoxide |
| 43 | Monocrotophos (mono) | Acaricide | Honeywell Riedel-de Haën (Seelze, Germany) | 99.9 | Methanol |
| 44 | Myclobutanil (myclob) | Fungicide | Institute of Organic Industrial Chemistry (Warsaw, Poland) | 99.1 | Ethanol |
| 45 | Nabam (nab) | Fungicide, herbicide | Honeywell Riedel-de Haën (Seelze, Germany) | 96.1 | Dimethyl sulfoxide/ water 1:10 (*V*/*V*) |
| 46 | Methyl parathion (para) | Insecticide | Dr. Ehrenstorfer (Augsburg, Germany) | 98.6 | Isopropanol |
| 47 | Pendimethalin (pen) | Herbicide | Dr. Ehrenstorfer (Augsburg, Germany) | 98.5 | Acetone |
| 48 | Phenmedipham (phen) | Herbicide | Dr. Ehrenstorfer (Augsburg, Germany) | 99.9 | Acetone |
| 49 | Phorate[a] (phor) | Insecticide | Dr. Ehrenstorfer (Augsburg, Germany) | 89.5 | Acetone |
| 50 | Picoxystrobin[a] (pico) | Fungicide | Dr. Ehrenstorfer (Augsburg, Germany) | n. s. | Methanol |
| 51 | Propineb (pro) | Fungicide | Honeywell Riedel-de Haën (Seelze, Germany) | n. s. | Toluene |
| 52 | Pymetrozine (pym) | Insecticide | Dr. Ehrenstorfer (Augsburg, Germany) | 99.0 | Ethanol |
| 53 | Pyriproxyfen (pyr) | Insecticide | Dr. Ehrenstorfer (Augsburg, Germany) | 99.0 | Methanol |
| 54 | Quinoxyfen (qui) | Fungicide | Honeywell Riedel-de Haën (Seelze, Germany) | n. s. | Acetone |
| 55 | Tebuconazole (teb) | Fungicide | Dr. Ehrenstorfer (Augsburg, Germany) | 98 | Isopropanol |
| 56 | Thiram (thi) | Fungicide | Honeywell Riedel-de Haën (Seelze, Germany) | 99 | Acetone |
| 57 | Thiacloprid (thia) | Insecticide | Dr. Ehrenstorfer (Augsburg, Germany) | 99.5 | Acetone |
| 58 | Triadimenol (tri) | Fungicide | Dr. Ehrenstorfer (Augsburg, Germany) | 98 | Acetone |
| 59 | Zineb (zin) | Fungicide | Fluka, Sigma-Aldrich (Steinheim, Germany) | 68.5 | Chloroform/ dimethyl sulfoxide 5:1 (*V*/*V*) |
| 60 | Ziram (zir) | Fungicide | UCB Chemical Sector (Brussels, Belgium) | 99.9 | Chloroform/ methanol 3:2 (*V*/*V*) |

n.s.: not specified; [a]liquid pesticide

**Table S2.** Pesticide concentration range investigated for estrogenic activity

| ID | Pesticide | Amount/zone [µg] | ID | Pesticide | Amount/zone [µg] |
|---|---|---|---|---|---|
| 1 | Acetamiprid | 0.002–25 | 31 | Iprovalicarb | 0.02–10 |
| 2 | Acephate | 0.02–10 | 32 | Isoproturon | 0.02–10 |
| 3 | Aldicarb | 0.02–10 | 33 | Malathion | 0.02–10 |
| 4 | Amitraz | 0.002–10 | 34 | Mancozeb | 0.002–10 |
| 5 | Atrazine | 0.02–10 | 35 | Maneb | 0.002–10 |
| 6 | Azoxystrobin | 0.002–10 | 35 | MCPA | 0.002–10 |
| 7 | Bentazon | 0.001–10 | 37 | Mecoprop | 0.02–10 |
| 8 | Carbendazim | 0.001–10 | 38 | Mercaptodimethur | 0.002–40 |
| 9 | Carbaryl | 0.02–40 | 39 | Metalaxyl | 0.02–10 |
| 10 | Chlorpyrifos | 0.02–10 | 40 | Metamitron | 0.02–10 |
| 11 | Chlorpropham | 0.001–10 | 41 | Metconazole | 0.001–10 |
| 12 | Cyfluthrin | 0.001–10 | 42 | Metiram | 0.002–10 |
| 13 | Cyprodinil | 0.02–50 | 43 | Monocrotophos | 0.02–10 |
| 14 | Cypermethrin | 0.02–70 | 44 | Myclobutanil | 0.02–10 |
| 15 | Dazomet | 0.002–10 | 45 | Nabam | 0.002–10 |
| 16 | Desmedipham | 0.001–10 | 46 | Methyl parathion | 0.02–10 |
| 17 | Difenoconazol | 0.001–10 | 47 | Pendimethalin | 0.002–20 |
| 18 | Diflubenzuron | 0.001–10 | 48 | Phenmedipham | 0.001–10 |
| 19 | Dimethomorph | 0.02–10 | 49 | Phorate | 0.02–10 |
| 20 | Dithianon | 0.001–10 | 50 | Picoxystrobin | 0.02–25 |
| 21 | Dpoxiconazol | 0.002–10 | 51 | Propineb | 0.002–1 |
| 22 | Fe(III)-dimethyldithio carbamate | 0.002–10 | 52 | Pymetrozine | 0.001–10 |
| 23 | Fenhexamid | 0.002–6 | 53 | Pyriproxyfen | 0.001–10 |
| 24 | Fenpropidin | 0.001–10 | 54 | Quinoxyfen | 0.001–10 |
| 25 | Fenpropimorph | 0.02–10 | 55 | Tebuconazole | 0.002–10 |
| 26 | Fludioxonil | 0.02–10 | 56 | Thiram | 0.002–10 |
| 27 | Folpet | 0.001–10 | 57 | Thiacloprid | 0.002–10 |
| 28 | Glyphosat | 0.002–10 | 58 | Triadimenol | 0.002–10 |
| 29 | Hexythiazox | 0.001–10 | 59 | Zineb | 0.002–10 |
| 30 | 2-Imidazolidinone | 0.002–10 | 60 | Ziram | 0.002–10 |

**Table S3.** List of 6 commercial PPPs and solutions prepared

| Plant protection product | Pesticide(s) | Manufacturer | Aggregate state | Concentration [µg/mL] | | |
|---|---|---|---|---|---|---|
| | | | | Stock solution in water | 1:10 dilution in methanol | 1:50 dilution in methanol |
| Dithane® NeoTec | Mancozeb | Indofil Industries (Amsterdam, Netherlands) | Solid | 750 | 75 | 15 |
| DYNALI® | Difenoconazol,<br>cyflufenamid | Syngenta (Basel, Switzerland) | Liquid | 60<br>30 | 6<br>3 | 1.2<br>0.6 |
| FOLPAN® 80 WDG | Folpet | Adama Deutschland (Cologne, Germany) | Solid | 800 | 80 | 16 |
| SWITCH® | Fludioxonil,<br>cyprodinil | Syngenta (Basel, Switzerland) | Solid | 250<br>375 | 25<br>37.5 | 5<br>7.5 |
| Teldor® | Fenhexamid | Bayer CropScience (Monheim am Rhein, Germany) | Solid | 500 | 50 | 10 |
| Vivando® | Metrafenone | BASF SE (Limburgerhof, Germany) | Liquid | 500 | 50 | 10 |

**Table S4.** PPP spray schedule used for the Rivaner white wine 2014 (Seiferling, Stettfeld, Germany)

| Spray no. | Date | Plant protection product | Concentration | Pesticide |
|---|---|---|---|---|
| 1 | 04/23/2014 | Dithane® NeoTec | 2.00 g/L | Mancozeb |
| | | sulphur | 2.00 g/L | Sulphur |
| 2 | 05/06/2014 | Dithane® NeoTec | 2.00 g/L | Mancozeb |
| | | sulphur | 9.00 g/L | Sulphur |
| 3 | 05/19/2014 | Dithane® NeoTec | 2.00 g/L | Mancozeb |
| | | sulphur | 9.00 g/L | Sulphur |
| 4 | 05/31/2014 | Folpan® 80 WDG | 1.00 g/L | Folpet |
| | | DYNALI® | 0.50 mL/L | Difenoconazol, diflufenamid |
| 5 | 06/12/2014 | Folpan® 80 WDG | 1.50 g/L | Folpet |
| | | DYNALI® | 0.75 mL/L | Difenoconazol, diflufenamid |
| 6 | 06/26/2014 | Folpan® 80 WDG | 1.00 g/L | Folpet |
| | | Vivando® | 0.20 mL/L | Metrafenone |
| | | SWITCH® | 0.60 g/L | Fludioxonil, cyprodinil |
| | | Mimic® | 0.50 mL/L | Tebufenozide |
| 7 | 07/07/2014 | Dithane® NeoTec | 2.00 g/L | Mancozeb |
| | | Systhane® 20 EW | 0.15 mL/L | Myclobutanil |
| 8 | 07/19/2014 | Ridomil Gold Combi | 1.50 g/L | Folpet, metaxalyl |
| | | Forum® Star | 1.20 g/L | Folpet, dimethomorph |
| | | Systhane™ 20 EW | 0.15 mL/L | Myclobutanil |
| 9 | 08/01/2014 | Forum® Star | 1.20 g/L | Folpet, dimethomorph |
| | | Vivando® | 0.20 mL/L | Metrafenone |
| | | Runner™ | 0.40 mL/L | Methoxyfenozide |
| 10 | 08/11/2014 | Funguran® progress | 1.25 g/L | Copper hydroxide |
| | | DYNALI® | 0.50 mL/L | Difenoconazol, diflufenamid |
| | | Vivando® | 0.20 mL/L | Metrafenone |
| | | Teldor® | 1.00 g/L | Fenhexamid |
| harvest | 09/20/2014 | - | - | - |

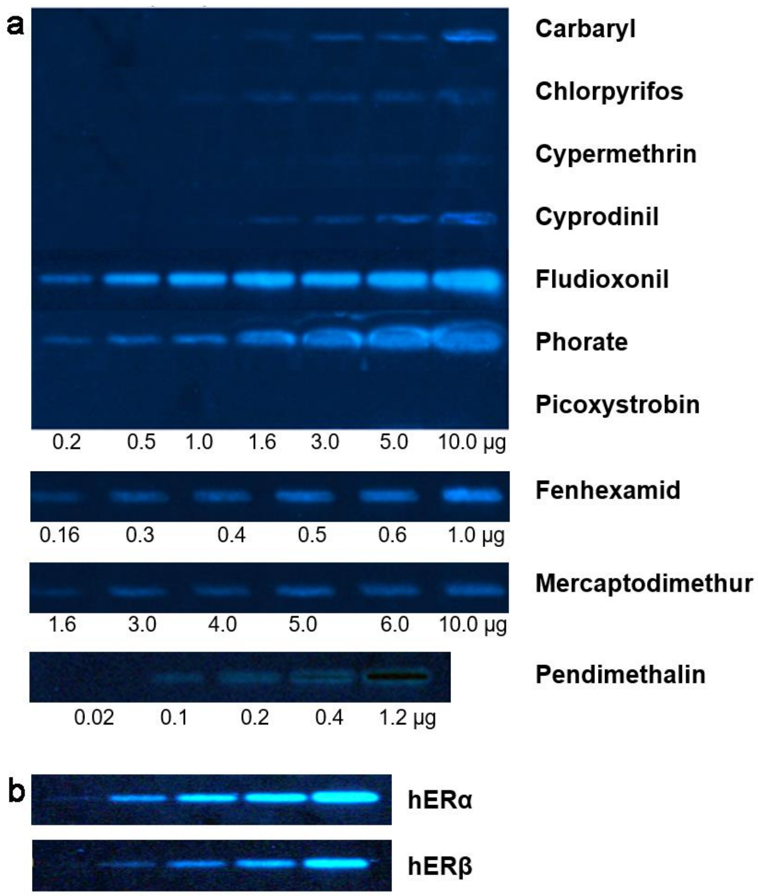


**Fig S1.** Planar estrogen screening of 10 pesticides applied at different amounts on the HPTLC plate silica gel 60 RP-18 W (without chromatography) showing plate images at FLD 366 nm with estrogenic pesticide zones observed as MU-blue fluorescent bands (**a**). Comparison of the responses of fenhexamid (100−600 ng/band) using the hERα receptor *versus* hERβ receptor (**b**), which shows that the latter has a weaker response.

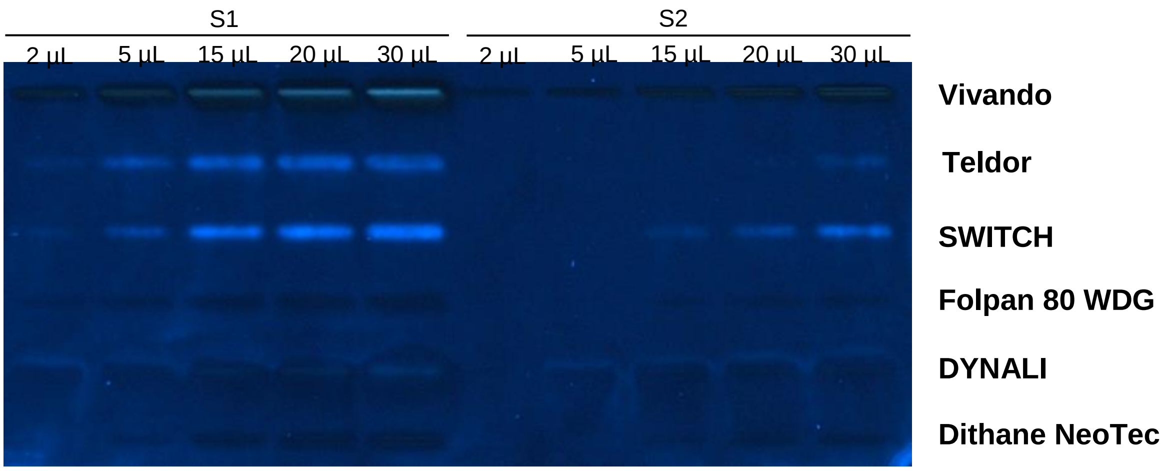


**Fig. S2.** Planar estrogenic screening of 6 PPPs (without chromatography) showing the bioautogram at FLD 366 nm with estrogen-like PPP zones as MU-blue fluorescent bands.

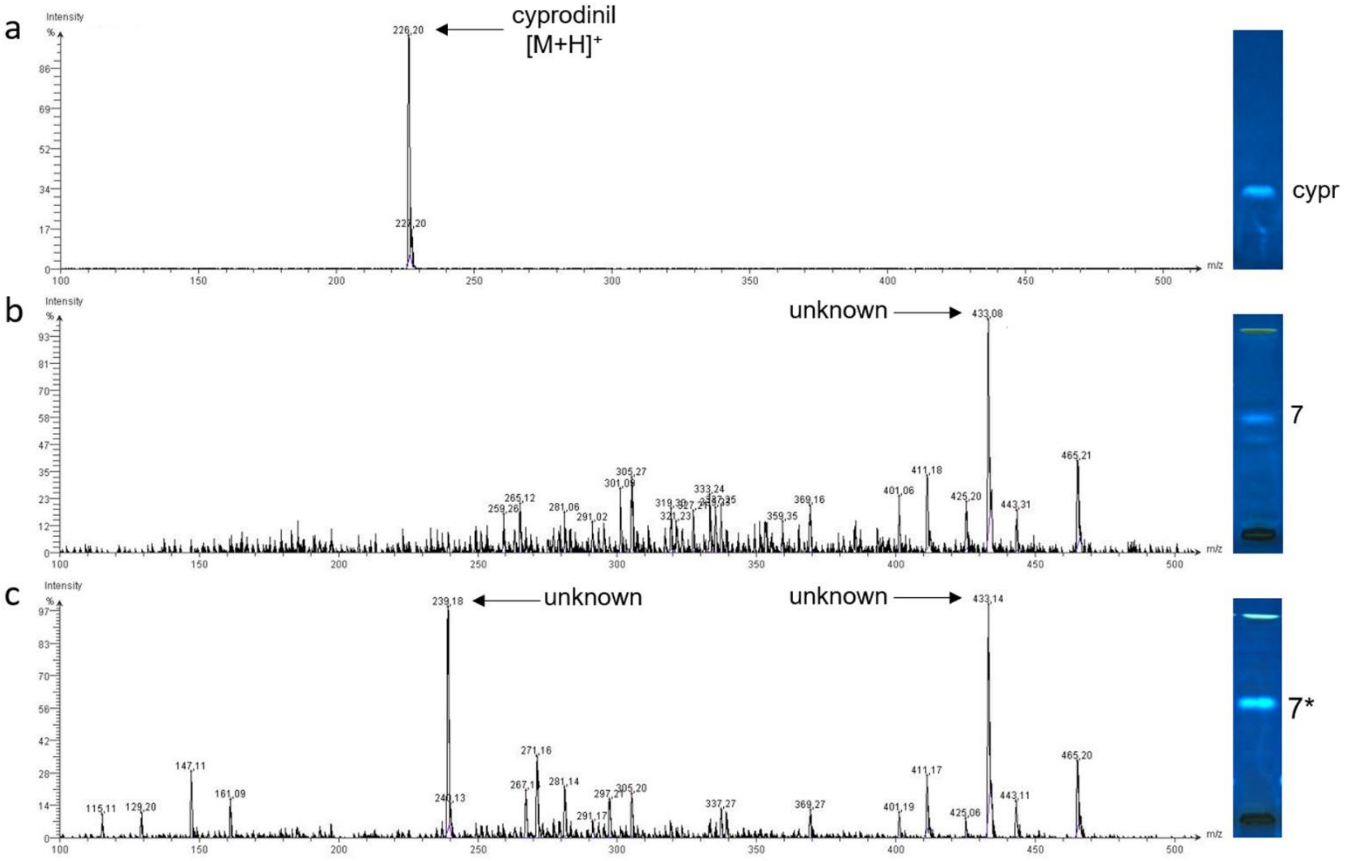


**Fig S3.** Mass spectra obtained via HPTLC–pYES–MS of the cyprodinil standard (**a**), zone **7** in tomato skin as negative control (**b**), and tomato skin extract treated with cyprodinil (**c**) containing the enhanced unknown zone **7***.
